# Study on Performance Testing and Evaluation of Autonomous Emergency Braking System Based on Self-Constructed Comprehensive Performance Evaluation Index Model

**DOI:** 10.3390/s25072171

**Published:** 2025-03-29

**Authors:** Dongying Liu, Wanyou Huang, Ruixia Chu, Zhenyu Li, Xiaoyue Jin, Hongtao Zhang, Yan Wang, Shaobo Ji

**Affiliations:** 1 Automotive Engineering College, Shandong Jiaotong University, Jinan 250357, China; liudongying2022@163.com (D.L.); churuixia@sdjtu.edu.cn (R.C.); 15306477529@163.com (Z.L.); 15834185062@163.com (X.J.); zht13293907672@163.com (H.Z.); sdzbgqwangyan@163.com (Y.W.); 2 Intelligent Testing and High-End Equipment of Automotive Power Systems, Shandong Province Engineering Research Center, Jinan 250000, China; jobo@sdu.edu.cn; 3 Jinan Engineering Research Center of Automotive Equipment and Technology, Jinan 250357, China; 4 College of Energy and Power Engineering, Shandong University, Jinan 250100, China

**Keywords:** AEB system, CPEIM, collision traffic accidents, typical test scenario, analytical hierarchy process

## Abstract

With the continuous development of assisted driving technology, the autonomous emergency braking (AEB) system has emerged as a critical innovation in preventing collisions and improving vehicular safety. In this paper, to test the performance of the AEB system efficiently and reliably in real-world driving scenarios, four typical test scenarios for the AEB system were constructed, and five comprehensive performance evaluation indices, including braking parking distance, braking deceleration, collision warning time, speed variation, and accident collision avoidance rate, were proposed for the first time. Subsequently, the Comprehensive Performance Evaluation Index Model (CPEIM) for the AEB system and scoring rules for typical test scenarios were established, which were applied to analyze data obtained from road testing, thereby enabling comprehensive testing and evaluation for AEB system performance. The results showed that the Tesla Model Y and Volvo S90 scored 1.8857 and 2.0433, respectively. Under conditions of dry pavement, across a range of test scenarios, the AEB system of both the Tesla Model Y and Volvo S90 were capable of averting collisions at speeds not exceeding 35 km/h and 45 km/h, respectively.

## 1. Introduction

**Figure 1:**
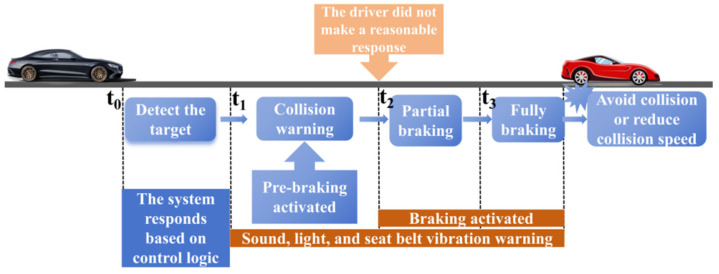
Working principle of the AEB system.

In the “Global Status Report on Road Safety 2023”, the World Health Organization (WHO) pointed out that about 1.19 million people died in road traffic accidents in 2021 [1]. Notably, drunk driving accounted for about 10% of fatal road traffic accidents, more than 50% were due to speeding. Distracted driving accounts for 14% to 31% of all motor vehicle collisions [2], and has been identified as the primary cause of accident by the WHO. The Advanced Driving Assistance System (ADAS) has been undergoing rapid development, aiming to significantly reduce traffic incident rates and enhance overall vehicular safety. The AEB is considered a promising active safety technology, and can minimize collision velocity or avoid collisions completely through vehicle warning and active braking interventions when the driver fails to respond in a timely manner under emergency situations. The working principle of AEB is shown in Figure 1. The research data from the American Highway Safety Insurance Association showed that the application of the AEB system in vehicles can lower traffic accidents by 27% [3]. The European and Australian New Car Assessment Programme (NCAP) stated that vehicles equipped with an AEB system can reduce rear end collisions by 38% when the speed was less than 50 km/h [4]. Active safety warning technologies in connected vehicle environments can effectively reduce collision probabilities by 17–34% through multi-dimensional risk perception and real-time collaborative decision-making [5].

As stated in the most recent study by Canalys, one-third of new vehicles in major markets, such as the Chinese Mainland, Europe, Japan, and the United States, were equipped with ADASs. The European Union (EU) mandated a requirement for M1 and N1 category vehicles in General Safety Regulation 2019/2144 (GSR 2019/2144): all new vehicles entering the EU market must be equipped with the AEB system functionality as defined in the United Nations Economic Commission for Europe Regulation 152 (UN ECE R152), effective from 6 July 2022 [6]. On 9 May 2024, the National Highway Traffic Safety Administration (NHTSA) officially promulgated Federal Motor Vehicle Safety Standard 127 (FMVSS 127), mandating that all newly manufactured vehicles available for commercial distribution must comply with specified performance requirements for AEB systems by September 2029 [7]. In the same year, the Ministry of Transport of China issued four drafts for soliciting opinions on the management of commercial vehicles, requiring commercial buses, cargo vehicles, towing vehicles, and dangerous goods commercial vehicles to be installed with AEB systems. The above regulatory frameworks adopted by these nations evidently demonstrate a growing recognition of AEB system performance evaluation as a critical safety priority. This technological assessment is evolving beyond pre-production sampling checks to become a mandatory component of periodic vehicle inspections in transportation safety protocols.

The operational stability and reliability of AEB systems are ensured through standardized testing protocols that have been established by global regulatory authorities and standardization bodies, mandating strict adherence to predetermined performance metrics as a prerequisite for vehicle homologation. In 2014, the assessment framework of Euro NCAP was enhanced through the incorporation of AEB system performance evaluation, with test scenarios being systematically categorized into three distinct operational conditions: stationary, moving, and braking vehicles within rear-end collision contexts [8,9]. The Australian NCAP evaluation standard for the AEB system included a total of 24 scores for pedestrian protection testing, with six scores each for bicycle testing and pedestrian testing [10]. The performance rating levels of AEB systems are divided into four categories (excellent, good, fair, and poor) by the Intelligent Vehicle Integrated Systems Test Area (i-VISTA) [11]. The Allgemeiner Deutscher Automobile Club (ADAC) accounted for 80% of the validity testing for AEB system performance ratings, including a reduction in speed and the first alarm time, each accounting for 50%.

The implementation of these regulatory standards has necessitated the execution of real-world vehicular testing under tripartite constraints: environmentally bounded parametric configurations, suboptimal cost-to-performance ratios, and operationally significant safety risks. Consequently, systematic optimizations of control algorithms and scenario-specific validation paradigms are being prioritized within global research communities to develop resource-efficient certification methodologies. Notably, contemporary evaluation architectures have incorporated multidimensional verification mechanisms, including multi-domain co-simulation platforms integrating crash probability models, naturalistic driving data mining techniques, hardware-in-the-loop (HIL) subsystem emulation, and vehicle-in-the-loop (VIL) integration frameworks. These advanced technical solutions have been methodologically embedded within standardized AEB assessment protocols to enhance testing reproducibility and scenario coverage.

Shu and He et al. [12,13], via MATLAB/Simulink and CarSim co-simulation, tested the effectiveness of the AEB model in existing test scenarios. Li [14] constructed typical intersection and standard regulatory test scenarios using PreScan 8.5.0, and combined Matlab/Simulink to build an AEB model based on time to collision (TTC) and perform virtual simulation tests on different collision types, such as vehicle–vehicle (V–V), vehicle–bicycle (V–B), and vehicle–pedestrian (V–P). A systematic review of AEB system validation methodologies was conducted through a critical analysis of representative studies. In seminal work by Jia et al. [15], a three-dimensional virtual test environment was constructed using 3D Studio Max, with a safety braking distance model subsequently developed through the integration of pedestrian psychological comfort parameters and vehicular deceleration dynamics. Parallel research efforts by Walter [16], David [17], Ren [18], and Yi [19] demonstrated that critical accident elements were extracted from traffic collision databases, enabling the reconstruction of prototypical accident scenarios. These scenarios were empirically validated through driving simulators, with particular emphasis on AEB system performance quantification under reconstructed collision conditions. Methodological advancements in AEB assessment were further evidenced by Song and Jiang et al. [20,21], where ADAS testbed instrumentation and differential global positioning systems were, respectively, implemented for compliance verification against established regulatory benchmarks. To enhance control algorithm verification efficiency, Xu et al. [22] proposed a co-simulation framework combining software-in-the-loop (SIL) and the HIL architectures for two-axle passenger vehicles. Experimental validation revealed that vehicular velocity and inter-vehicle distance measurements obtained from SIL simulations exhibited ≤ 2.3% deviation from HIL counterparts. Substantial progress in the VIL methodologies was documented by Lu, Gao, and Fang et al. [23,24,25,26]. Notably, a sensor-injection-based VIL platform was developed by Gao et al. [25], wherein virtual sensor signals were systematically integrated with physical vehicle dynamics. Comparative analyses demonstrated that longitudinal acceleration profiles and collision avoidance success rates obtained through this platform exhibited strong congruence (R^2^ = 0.93) with real-world road testing data.

Based on the aforementioned analysis, the existing evaluation systems are constrained by the following limitations:

- Current performance evaluation methodologies for AEB systems are predominantly based on simplified or idealized scenarios (e.g., fixed speeds, favorable weather, and optimal lighting conditions), which fail to account for uncertainties inherent in real-road environments, such as dynamic traffic participants and complex environmental interactions.
- Existing studies isolate scenario construction, system performance, and evaluation metrics, lacking an integrated analytical framework. This fragmentation results in evaluation outcomes that inadequately reflect the comprehensive performance of the system.
- While the HIL and VIL testing enable diverse scenario generation, their reliance on specialized equipment and compatibility with specific vehicle models leads to high development costs and limited scalability. Meanwhile, driving simulator-based evaluations are compromised by subjective interference.
- With the proliferation of L2+/L3 autonomous driving systems, the AEB system is challenged to address increasingly complex scenarios (e.g., pedestrian crossings), for which traditional evaluation methods prove insufficient in verifying advanced functionalities.

To address these challenges, this study made the following advancements: (1) Typical test scenarios were constructed based on authentic collision accident data, providing a scenario library closely aligned with real-road conditions. This approach addresses the deficiency in scenario coverage under existing standards (e.g., E-NCAP and i-VISTA) and enhances the practical relevance of evaluations. (2) A comprehensive assessment framework was developed by integrating safety, reliability, and comfort metrics, overcoming the limitations of single collision-avoidance indices. This framework advances AEB system evaluation from a “single-dimensional collision avoidance” paradigm to a “holistic performance assessment” paradigm. (3) A novel AEB system performance evaluation model was established by integrating scenarios, system parameters, and evaluation criteria into a cohesive structure. In an innovative approach, test scenarios were designated as the standards layer, while evaluation indices were assigned as the scheme layer, ensuring the CPEIM. (4) An efficiency-optimized hybrid methodology combining co-simulation (MATLAB/Simulink 2020a-PreScan 8.5.0) with real-vehicle testing was proposed. (5) The proposed model was validated through road tests conducted with a Tesla Model Y and a Volvo S90 under representative scenarios. The results confirm the model’s efficacy and establish a new paradigm for the comprehensive evaluation of AEB system performance.

## 2. Construction of Typical Test Scenarios

### 2.1. Extraction of Accident Data Element

**Figure 2:**
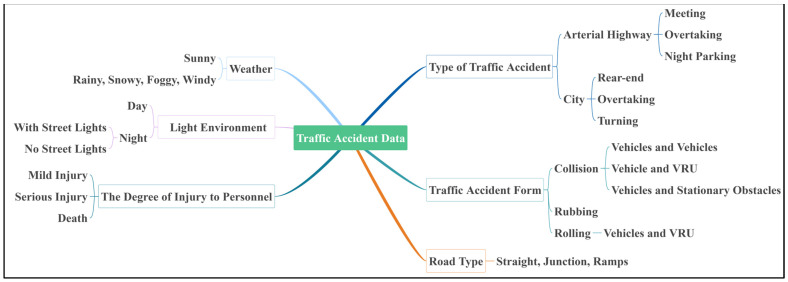
Types of traffic accident data.

The traffic accident data were obtained from the Judicial Expertise Center of Shandong Jiaotong University (hereinafter referred to as “the Center”), comprising the annual records for the year 2020 from the cities of Jinan and Tai’an in Shandong Province, China. The dataset covers road environments categorized as urban roadways, urban–rural transitional zones, and rural township roads. The traffic accident data are shown in Figure 2.

To establish a structured framework, during the scenario selection phase, scenario elements were organized into eight parametric dimensions: weather condition, time and location, main vehicle motion state, target type, target motion state, relative movement with the main vehicle, road type, and road classification; 146 accident scenarios were systematically parameterized according to the predefined criteria outlined in Table 1. The manual extraction of accident scene characteristics was conducted, followed by a rigorous analysis of primary accident features. Similar scenarios were aggregated into homogeneous clusters under standardized classification protocols (e.g., rainy and snowy conditions were reclassified as adverse weather, while three-wheeled and two-wheeled electric vehicles were consolidated into the two-wheeled vehicle category when other variables remained invariant). Therefore, 25 distinct scenario clusters were ultimately derived, including the following:

- Intersectional V–P perpendicular conflicts.
- Straight-road V–P collisions (co-directional/opposing) under dry/rainy conditions.

The statistical distribution of scenario element constituent ratios derived from the accident database are presented in Table 2, forming a quantitative framework for AEB performance test scenarios.

### 2.2. Construction of Scenario

Due to the numerical characteristics and sample size constraints, the K-means clustering method was employed using SPSS (v27) software to categorize the 25 accident scenario clusters. The validity of the clustering outcomes was systematically verified through a comprehensive analysis of the final cluster centroid, inter-cluster centroid distance, ANOVA table, and performance metrics including between-/within-group variance ratios (F-values) and statistical significance (*p*-values). The final cluster centroids and their pairwise distances are presented in Table 3 and Table 4.

By synthesizing the data from Table 1 and Table 3, the 25 accident scenario clusters were classified into four major categories:

- Collisions between motor vehicles and vulnerable road users (VRUs) at urban road intersections under adverse daytime weather conditions.
- Collisions between motor vehicles traveling straight and VRUs moving in the same direction on straight rural road segments during nighttime without street lighting.
- Collisions involving VRUs crossing urban road intersections and motor vehicles under favorable daytime weather conditions.
- Rear-end collisions between motor vehicles on highways under adverse daytime weather conditions.

As evidenced by Table 4, substantial inter-cluster centroid distances were observed, indicating strong inter-cluster separation and high clustering quality.

To validate the clustering results, the ANOVA table (Table 5) was critically analyzed. Key metrics including F-values (representing between-group variance ratios), *p*-values (statistical significance), and effect sizes (η^2^, quantifying variable contributions to group differences) were systematically evaluated to confirm the robustness of the classification framework.

As demonstrated in Table 5, all variables were found to exhibit statistically significant contributions to cluster differentiation (*p* < 0.001), confirming that the clustering results effectively captured the distinct characteristics of the selected variables. Road classification (F = 72.74, η^2^ = 0.913), relative motion to the host vehicle (F = 58.32, η^2^ = 0.894), and road type (F = 56.37, η^2^ = 0.882) demonstrated exceptionally high effect sizes, identifying them as the most critical features defining cluster boundaries. While target object motion state (η^2^ = 0.484) and weather conditions (η^2^ = 0.509) exhibited relatively lower contributions, their statistical significance was still confirmed (*p* < 0.001).

These results collectively validate that the clustering framework successfully identified critical discriminative patterns in traffic accident scenarios. The methodology is thus deemed statistically robust for generating representative test scenarios that encapsulate real-world collision dynamics.

By means of a multivariate statistical analysis of operational scenario clusters across different categories based on their distinctive characteristics, representative test scenarios were constructed, as presented in Table 6. The four identified accident categories exhibit highly distinct causal patterns:

- Category 1: Highway rear-end collisions under adverse weather conditions. This category is defined by rear-end collisions between motor vehicles, which are primarily attributed to the combined effects of elevated vehicle speeds and reduced road adhesion coefficients. These factors are observed to collectively contribute to delayed braking responses and extended stopping distances during sudden emergency braking scenarios, ultimately resulting in collisions.
- Category 2: Urban/Rural intersection conflicts. High-frequency collision risks across urban, provincial/national, and township road networks are characterized by complex interactions among multiple risk factors. These include dense traffic participant volumes, frequent intersection encounters with VRUs, and visual obstructions caused by vegetation or architectural structures. Such conditions are identified as key contributors to vehicle–VRU (V–VRU) accidents.
- Category 3: Sensor degradation-induced incidents. Accidents within this classification are caused by adverse weather conditions that simultaneously degrade sensor perception capabilities and reduce road adhesion coefficients. This dual effect is demonstrated to induce significant inaccuracies in collision warning system timing, thereby compromising vehicle safety margins. Failure to detect pedestrians or cyclists in a timely manner is shown to result in V–VRU collisions.
- Category 4: Nighttime rural road collisions. This category involves collisions predominantly occurring on unlit rural roads under nighttime conditions. Hazardous driving environments are created by compromised driver visibility and inadequate roadway illumination, which are found to collectively increase the likelihood of collisions between motor vehicles and pedestrians.

## 3. Construction of the Performance Evaluation Framework

The performance evaluation indices of the AEB system were analyzed in combination with typical accident scenarios. The weights of these evaluation indices were determined using the analytic hierarchy process (AHP), and a comprehensive performance evaluation model for the AEB system was subsequently established. Based on existing regulations and accident data, scoring criteria under various operational conditions were formulated across typical test scenarios, thereby providing systematic guidelines for the comprehensive assessment of AEB system performance.

### 3.1. Determination of Evaluation Indices

Through a systematic analysis of real-world accident data, three critical parameters were identified as essential considerations: braking characteristics, accident casualty severity, and driving comfort. Building upon these findings, a novel CPEIM was developed to assess the performance of AEB systems. The evaluation framework was constructed based on five rigorously defined indices: braking parking distance, braking deceleration, collision warning time, speed variation, and accident collision avoidance rate. These indices enable the precise quantification of AEB system effectiveness via multidimensional performance analysis. The operational definitions and measurement protocols for each evaluation index are elaborated in the subsequent sections as follows:

1. The brake parking distance of the testing vehicle.

The brake parking distance (*s*) is the distance traveled by a vehicle from the moment the driver suddenly steps on the brake pedal at a certain initial speed until the vehicle comes to a complete stop. The calculation equation is as follows:

(1) $$s=v_{x}\left(t_{d}+\frac{t_{s}}{2}\right)+\frac{{v_{x}}^{2}}{2\mu g},$$

where *s* is the brake parking distance (m), *v_x_* is the speed of the tested vehicle when braking (m/s), *t*_d_ is the total system delay time (s), including sensor, decision, and execution delays, *t*_s_ is the braking force rise time (s), *μ* is the road adhesive coefficient (-), and g is the gravity acceleration (m/s^2^).

According to Equation (1), the influencing factors of vehicle braking distance mainly include vehicle speed, road adhesion coefficient, and reaction time. In emergency situations, the driver or AEB system applies emergency braking and the smaller the parking distance, the greater the possibility of collision avoidance.

2. The braking deceleration.

The braking deceleration was recorded as *a_b_*, which refers to the mean fully developed deceleration (*MFDD*) when the vehicle speed drops from 0.8 times the initial speed to 0.1 times. The maximum braking deceleration is related to the road adhesion coefficient. The *MFDD* is an important parameter for evaluating the safety and comfort of AEB systems, as shown in Equation (2).

(2) $$MFDD=\frac{{v_{b}}^{2}-{v_{e}}^{2}}{25.92(S_{b}-S_{e})},$$

where *v_b_* is 0.8 times the initial vehicle speed (km/h), *v_e_* is 0.1 times the initial vehicle speed (km/h), *S_b_* is the distance traveled when the initial vehicle speed drops to 0.8 times (m), and *S_e_* is the distance traveled when the initial vehicle speed drops to 0.1 times (m).

3. The collision warning time of AEB system model.

The collision warning time was recorded as *T*_c_ (s); it refers to the time from the AEB system detecting a collision risk and issuing a warning to intervening in braking, which is related to the sensor type, detection range, and control strategy. It can be determined as the first step of AEB system intervention and is an important index for assessing the reliability, stability, and safety of the AEB system.

4. The speed variation.

The speed variation (Δ*v*) refers to the difference between the vehicle speed when the AEB system starts to intervene in braking and after stopping, as shown in Equation (3). Vehicle stopping includes two types: successfully avoiding collision and reducing the speed to 0 km/h, and colliding with the target object. The collision speed directly determines the degree of accident casualties. For every 5% decrease in vehicle speed variation rate, the accident fatality rate diminishes by 30% [27].

(3) $$\Delta v=v_{0}-v_{1},$$

where Δ*v* is the speed variation (km/h), *v*_0_ is the initial speed of the tested vehicle (km/h), and *v*_1_ is the collision speed or 0 (km/h).

5. Accident collision avoidance rate.

Accident collision avoidance rate was recorded as *R*_c_; it refers to the probability of vehicles equipped with an AEB system to avoid collision accidents, which is the basic index to measure the reliability of an AEB system. At present, accident avoidance rate detection is mainly used to optimize the feasibility of AEB system control strategy.

### 3.2. Determination of Evaluation Indices Weight

The CPEIM for AEB systems was developed utilizing the AHP. The hierarchical structure comprises three layers: (1) the target layer, defined as the CPEIM of the AEB system; (2) the standard layer, categorized into four typical test scenarios; and (3) the scheme layer, incorporating five critical evaluation indices. The detailed hierarchical framework and corresponding indices are shown in Table 7.

The relative weights between the standard layer and the target layer in the proposed hierarchical model were determined using the Saaty proportional scaling method [28]. This weighting process was conducted by integrating three critical accident-related parameters: (1) the statistical probability of traffic accidents, (2) the proportional distribution of scenario-specific elements, and (3) the severity of personnel injuries. The important relationships of AEB system test scenarios are summarized in Table 8.

Matrix **A** can be judged from Table 8:

(4) $$A=\left[\begin{array}{cccc} 1 & 1/3 & 1/4 & 1/2\\ 3 & 1 & 1/2 & 2\\ 4 & 2 & 1 & 3\\ 2 & 1/2 & 1/3 & 1 \end{array}\right],$$

The **w***_i_* was calculated by the square root method, and then the m-dimensional vector **w***_m_* was obtained, which was standardized to obtain the weight vector **w***_i_*. There are shown in Equations (5) and (6).

(5) $$w_{m}=\left[\begin{array}{c} 0.4518\\ 1.3161\\ 2.2134\\ 0.7598 \end{array}\right],$$

(6) $$w_{i}=\left[\begin{array}{c} 0.0953\\ 0.2776\\ 0.4668\\ 0.1603 \end{array}\right],$$

The maximum eigenvalue λ_max_ was calculated by Equation (7):

(7) $$\lambda \max=\frac{1}{4}\sum_{i=1}^{4}\frac{\left(Aw_{i}\right)}{w_{i}}=4.0311,$$

A consistency check was conducted. Since the judgment matrix **A** is of fourth order, by searching for random one-time indices, the random index (*RI*) was taken as 0.90. The consistency check is shown in Equations (8) and (9).

(8) $$CI=\frac{\lambda_{\max}-n}{n-1}=\frac{4.0311-4}{3}=0.0104,$$

(9) $$CR=\frac{CI}{RI}=\frac{0.0104}{0.90}=0.0116<0.1,$$

where *CR* is the consistency ratio (-).

The consistency check passed, indicating that the consistency level of the judgment matrix **A** is within the allowable range. Therefore, the weight matrix **w***_i_* calculated above was reasonable.

Judgment matrices corresponding to the scheme layer were systematically formulated for each test scenario within the criterion layer. Subsequently, the relative weights of individual evaluation indices in the scheme layer were quantitatively assessed across four typical test scenarios. Based on the independently constructed scheme layer, judgment matrices **B**, **C**, **D**, and **E** for the relationship importance tables for each typical test scenario were obtained. The relationship importance tables are shown in Appendix A, Table A1, Table A2, Table A3 and Table A4. The judgment matrices are shown in Equations (10)–(13):

(10) $$B=\left[\begin{array}{ccccc} 1 & 2 & 1/2 & 2 & 1/3\\ 1/2 & 1 & 1/3 & 1 & 1/3\\ 2 & 3 & 1 & 3 & 1/2\\ 1/2 & 1 & 1/3 & 1 & 1/3\\ 3 & 3 & 2 & 3 & 1 \end{array}\right],$$

(11) $$C=\left[\begin{array}{ccccc} 1 & 2 & 1/3 & 3 & 1/3\\ 1/2 & 1 & 1/4 & 2 & 1/4\\ 3 & 4 & 1 & 4 & 1/2\\ 1/3 & 1/2 & 1/4 & 1 & 1/5\\ 3 & 4 & 2 & 5 & 1 \end{array}\right],$$

(12) $$D=\left[\begin{array}{ccccc} 1 & 3 & 1/3 & 2 & 1/3\\ 1/3 & 1 & 1/3 & 2 & 1/4\\ 3 & 3 & 1 & 3 & 1/2\\ 1/2 & 1/2 & 1/3 & 1 & 1/5\\ 3 & 5 & 2 & 5 & 1 \end{array}\right],$$

(13) $$E=\left[\begin{array}{ccccc} 1 & 3 & 1/3 & 3 & 1/2\\ 1/3 & 1 & 1/4 & 2 & 1/5\\ 3 & 4 & 1 & 5 & 1\\ 1/3 & 1/2 & 1/5 & 1 & 1/5\\ 2 & 5 & 1 & 5 & 1 \end{array}\right],$$

The weight vectors **w***_iB_*, **w***_iC_*, **w***_iD_*, and **w***_iE_* of each index are shown in Equations (14)–(17).

(14) $$w_{iB}=\left[\begin{array}{c} 0.1585\\ 0.0965\\ 0.2668\\ 0.0965\\ 0.3817 \end{array}\right],$$

(15) $$w_{iC}=\left[\begin{array}{c} 0.1447\\ 0.0901\\ 0.2962\\ 0.0603\\ 0.4087 \end{array}\right],$$

(16) $$w_{iD}=\left[\begin{array}{c} 0.1484\\ 0.0903\\ 0.2709\\ 0.0710\\ 0.4194 \end{array}\right],$$

(17) $$w_{iE}=\left[\begin{array}{c} 0.1691\\ 0.0790\\ 0.3537\\ 0.0573\\ 0.3409 \end{array}\right],$$

A rigorous consistency validation process was implemented, with *RI* assigned a reference value of 1.12 and *CR* remaining below the 0.1 threshold. This successful verification confirmed the methodological validity of the weight assignments across all test scenarios, demonstrating their statistical appropriateness for subsequent analytical applications.

### 3.3. Construction of the CPEIM

The weights assigned to individual test scenarios and evaluation indices were calculated through the AHP, thereby providing a theoretical foundation for establishing the CPEIM of the AEB system. The calculated weight coefficients are systematically presented in Table 9 and Table 10.

Through the aforementioned analysis, the CPEIM for the AEB system was established, with its mathematical formulation explicitly defined in Equation (18). The proposed evaluation model encompassed five critical performance indices: braking parking distance, braking deceleration, collision warning time, speed variation, and accident collision avoidance rate. These indices were systematically integrated with four fundamental operational parameters: road adhesion coefficient, relative vehicle speed, braking coordination time, and relative deceleration, to establish a comprehensive multidimensional evaluation framework. Through this integrated parameter-index architecture, the model facilitates a rigorous assessment of three core AEB system attributes: operational safety, functional reliability, and dynamic stability under varying operational conditions.

(18) $$\begin{array}{c} CPEIM=0.0953\times (0.1585s+0.0965a_{b}+0.2668T_{c}+0.0965\Delta v+0.3817R_{c})\\ +0.2776\times (0.1447s+0.0901a_{b}+0.2962T_{c}+0.0603\Delta v+0.4087R_{c})\\ +0.4668\times (0.1484s+0.0903a_{b}+0.2709T_{c}+0.0710\Delta v+0.4194R_{c})\\ +0.1603\times (0.1691s+0.0790a_{b}+0.3537T_{c}+0.0573\Delta v+0.3409R_{c}) \end{array},$$

### 3.4. Establishment of Scoring Standards

The different performance evaluation standards for the AEB system directly affect the final evaluation results. Given the inherent complexities arising from multi-dimensional evaluation metrics and complex influencing factors associated with AEB system, scenario-specific evaluation protocols and scoring methodologies were formulated by integrating the CPEIM, referencing established regulatory frameworks, and adapting to distinct test scenario configurations. As a representative illustration, the performance scoring criteria for the AEB system at varying vehicle speeds in test scenario 2 are summarized in Table 11. The scoring criteria for other typical test scenarios are shown in Table A5, Table A6 and Table A7 of Appendix A.

The scoring criteria of braking parking distance refer to the 2018 i-VISTA autonomous vehicle challenge rules designed by the China Automotive Engineering Research Institute and are shown in Table 12.

The average braking deceleration is closely related to fuel economy and driving comfort and the smaller the MFDD, the better, when ensuring collision avoidance. According to the Chinese Standard GB 7258-2017 “Technical Conditions for Safety of Motor Vehicle Operation” [29] and the braking deceleration size specified in EU regulation 2009/40/EC, the scoring criteria were formulated, with full marks for collision avoidance and half for non-avoidance, as shown in Table 13.

According to Table 11, Table 12 and Table 13, the AEB system performance evaluation criteria were observed to exhibit significant inconsistencies across diverse test scenarios and operational conditions. By employing a systematic application of the CPEIM framework coupled with multidimensional evaluation criteria, this study established a quantitative scoring mechanism that supported the comprehensive assessment of AEB system performance under both isolated and combined test scenarios. The derived performance metric demonstrates positive correlation characteristics, where elevated scores correspond to enhanced system effectiveness. This analytical approach facilitates the development of standardized scoring protocols through CPEIM-based parametric modeling, thereby providing a theoretical foundation for identifying and prioritizing key performance-influencing factors in AEB system optimization.

## 4. Co-Simulation

A co-simulation framework integrating PreScan 8.5.0 (for scenario visualization and sensor modeling) with MATLAB/Simulink 2020a (for control logic implementation) was developed to establish high-fidelity vehicle dynamics models, hierarchical control strategy architectures for the AEB system, and test scenarios. The performance of the AEB system was tested by varying the influencing factors such as the vehicle speed, load capacity, sensor parameters, and road adhesion coefficient of the testing vehicle. Furthermore, on the basis of the CPEIM, the key influencing factors of the AEB system were analyzed to establish a foundation for developing effective testing conditions.

### 4.1. Model Building

The model parameters of the AEB system are summarized in Table 14. The control logic is divided into three sequential stages:

- Key parameters (including the relative distance, velocity, and azimuth angle of the detected target) are detected by front-mounted sensors.
- Collision prediction is performed through the TTC calculations (as defined in Equation (19)).
- Brake intervention is triggered when the TTC value falls below the predefined threshold.

**Figure 3:**
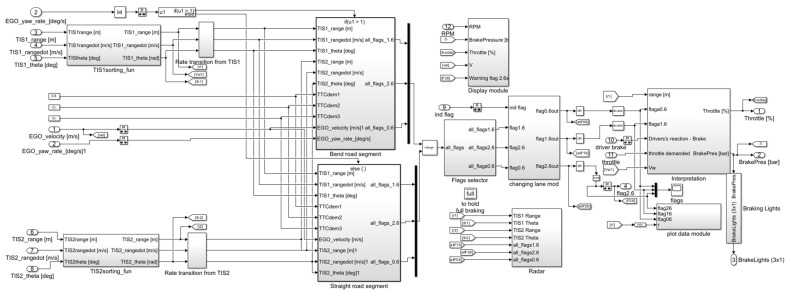
The AEB system control strategy.

**Figure A1:**
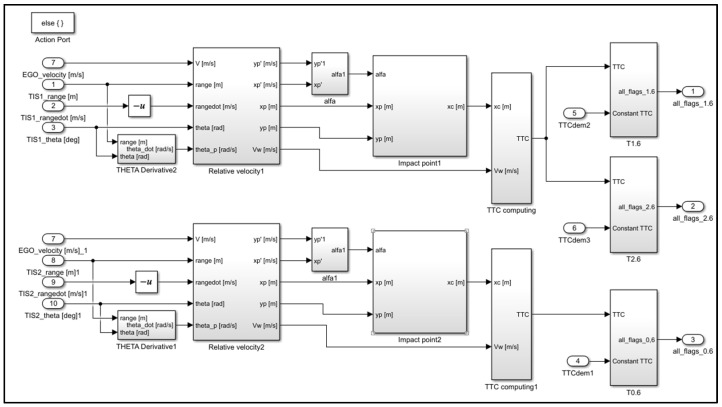
The straight path control model.

**Figure A2:**
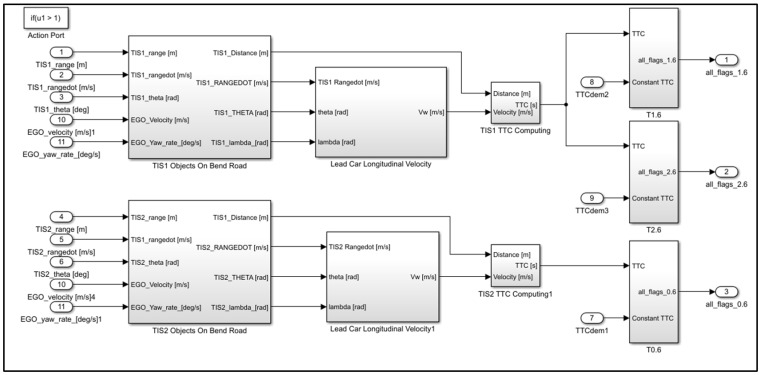
The bend path control model.

**Figure A3:**
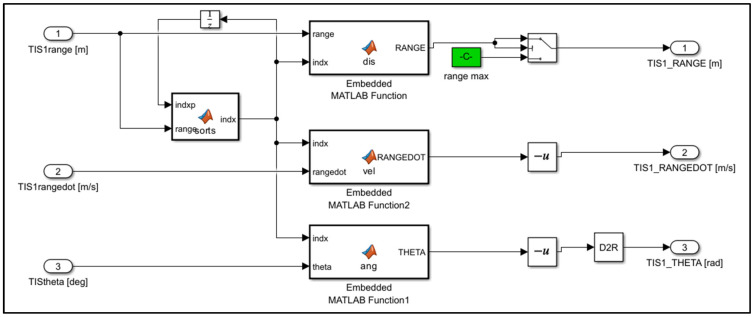
The sensor signal processing model.

**Figure A4:**
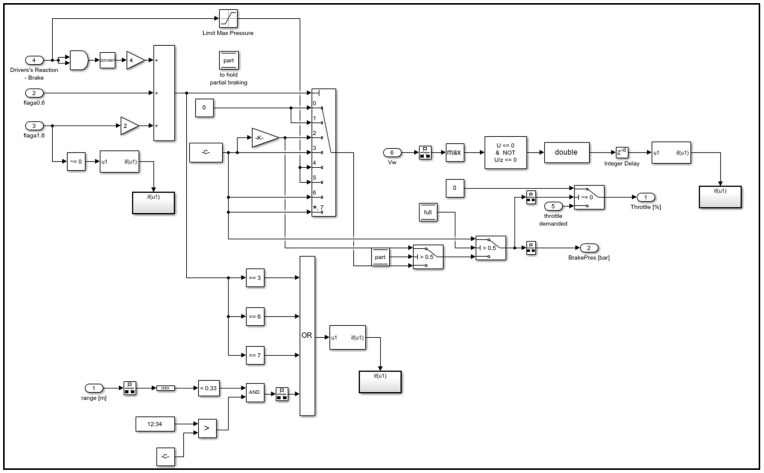
The brake intervention control model.

**Figure A5:**
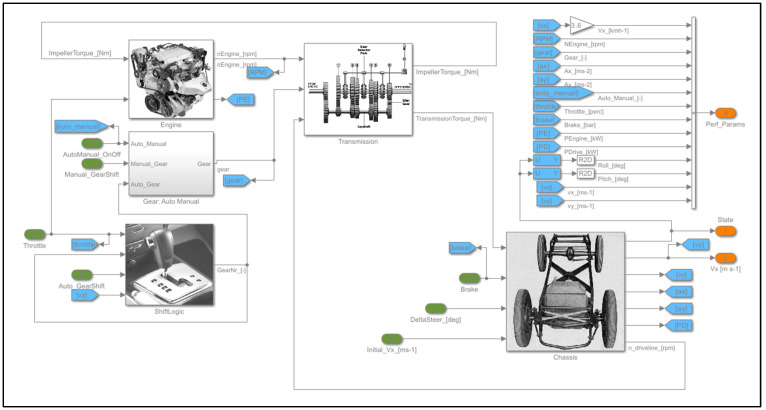
The vehicle dynamics model.

Four typical test scenarios were constructed in PreScan, and co-simulation tests were conducted to quantitatively evaluate the influence of critical factors (vehicle speed, road adhesion coefficient, relative distance, vehicle load, and sensor configurations) on AEB performance. The architecture of the AEB system model is illustrated in Figure 3, with vehicle dynamics model parameters detailed in Table 15. Subsystem modules, including straight and bend path control, sensor signal processing, brake intervention control, and vehicle dynamics modeling, are schematically represented in Figure A1, Figure A2, Figure A3, Figure A4 and Figure A5 of Appendix B.

(19) $$TTC=\frac{\Delta d}{\Delta v_{r}}$$

where Δ*d* is the relative distance between the main vehicle and target object (m), and Δ*v*_r_ is the relative speed between the main vehicle and target object (km/h).

### 4.2. Data Analysis

As an active collision avoidance technology, the operational reliability and safety of the AEB system were governed by some fundamental determinants: vehicle braking performance, vehicle speed, onboard sensor configuration, road adhesion conditions, lighting environment, control strategies, etc. A systematic investigation was conducted by integrating real-world vehicular operational states, standardized driving cycles, and typical driving scenarios, with focused parameterization of four critical variables: vehicle speed, road adhesion coefficient, vehicle load, and sensor configuration. The CPEIM enabled the quantitative characterization of these critical parameter interactions and established a standardized framework. This methodological approach facilitated the acceleration of AEB system development cycles while ensuring the comprehensive security verification of performance.

#### 4.2.1. Speed and Road Adhesion Coefficient

**Figure 4:**
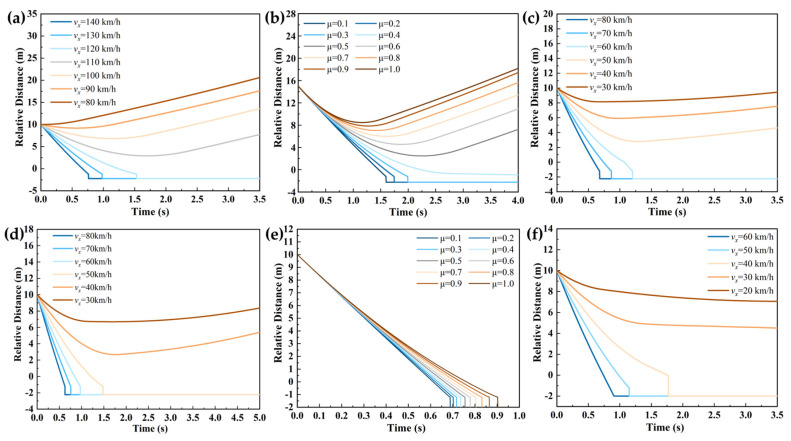
Performance testing results of the AEB system under different speeds and road adhesion coefficients. The simulation results of testing conditions 1–6 are shown in (**a**), (**b**), (**c**), (**d**), (**e**), and (**f**), respectively.

Four typical test scenarios were designed with primary variables including ego vehicle speed, target vehicle speed, relative distance, and road adhesion coefficient. As systematically outlined in Table 16, the simulation matrix allocated vehicle speed as the dominant variable in scenarios 1, 3, 4, and 6, whereas road adhesion coefficient served as the principal variable in scenarios 2 and 5. The resultant performance metrics across these parameterized scenarios are comparatively visualized in Figure 4, revealing speed-dependent response patterns and adhesion-limited braking behaviors.

Collisions are typically initiated when relative distance thresholds are ≤ 0 m. As demonstrated in Figure 4, collision avoidance failures were observed across six simulation scenarios, revealing the critical influence of velocity parameters and road adhesion coefficients on AEB system safety and reliability in conventional driving conditions. This phenomenon primarily stems from extended braking distances caused by either increased velocity or reduced adhesion coefficients. Notably, the road adhesion coefficient directly governs maximum achievable braking deceleration, thereby determining vehicular safety margins and dynamic stability. Under emergency braking conditions, diminished relative distances were found to critically compress system warning intervals and intervention windows, ultimately resulting in collision mitigation failures under conventional AEB control strategies.

**Figure 5:**
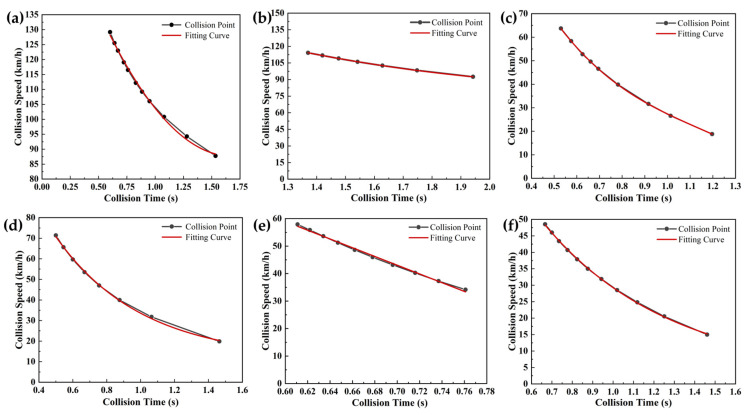
Fitting data under different testing conditions: The fitting results of collision speeds for simulation testing conditions 1–6 are shown in (**a**), (**b**), (**c**), (**d**), (**e**), and (**f**), respectively.

To facilitate the quantitative assessment of velocity and road adhesion coefficient effects on AEB system performance across operational scenarios, collision velocity parameters were systematically fitted for each testing condition. As presented in Figure 5, the experimental results demonstrated strong dependencies between AEB system safety performance and two critical parameters: vehicle speed and road adhesion coefficient. A linear correlation between post-braking collision speeds was observed under minor velocity intervals (Δ*v* < 15 km/h). These findings provide critical insights for refining AEB system control strategies, suggesting that scenario-specific activation thresholds should be established according to real-time speed and adhesion conditions to enhance both safety performance and adaptive capabilities of the system. The fitting equations are shown in Table A8 of Appendix A.

#### 4.2.2. Vehicle Load

**Figure 6:**
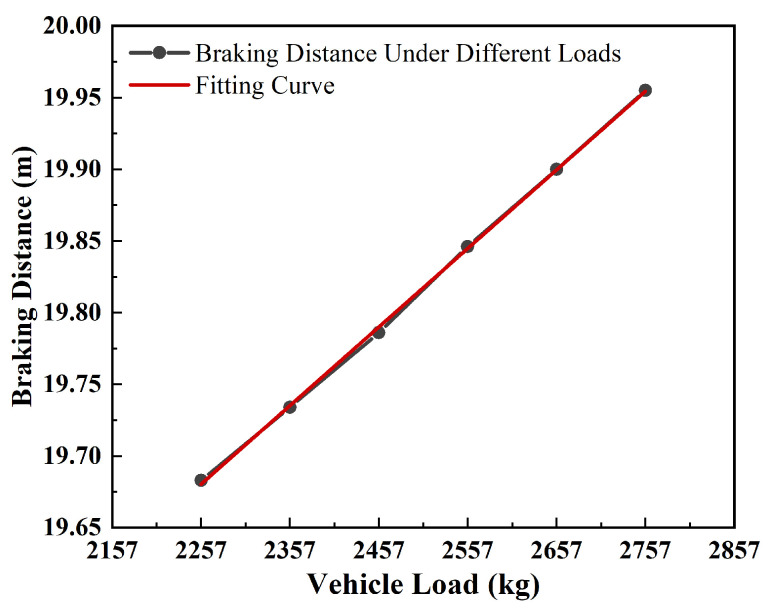
The variation law of braking distance with vehicle load.

A seven-seat SUV was selected as the research object with an initial spring-loaded mass of 2257 kg, that was increased to 3257 kg with a step of 100 kg when conducting the co-simulation. The test scenario involved the testing vehicle traveling at a constant speed of 60 km/h on a road surface with an adhesion coefficient of 0.85, and a stationary obstacle was positioned 20 m ahead at time zero. The results showed that the collision was successfully avoided at 2.245 s when the load was 2257 kg; the vehicle could not avoid collision under the same control strategy and driving conditions when the load was 2857 kg, and collided with an obstacle ahead at 2.196 s. The simulation data were extracted and analyzed, with quantitative variations in braking distance across vehicle load configurations systematically documented in Figure 6.

As evidenced in Figure 6, a progressive elongation of braking distances was observed with increasing vehicular load magnitudes, exhibiting linear proportionality between mass increments and braking distance extensions. Under emergency braking conditions, predefined AEB algorithms were found insufficient to ensure collision mitigation for fully loaded vehicle configurations, consequently compromising system safety margins. This operational limitation was particularly evident in heavy-duty truck applications where mass variations between unloaded and fully loaded states exceeded conventional design parameters. These findings necessitate that AEB control algorithm development systematically incorporates three critical factors: road adhesion coefficient variations, velocity-dependent response thresholds and mass-dependent braking dynamics across loading conditions.

#### 4.2.3. Sensor Configuration

Contemporary vehicular perception systems predominantly employ radar and vision-based sensors, and the multi-sensor fusion frameworks gain prominence in intelligent connected vehicles for robust object detection and classification. To systematically evaluate sensor configuration impacts on AEB performance, a parametric simulation study was conducted across four critical sensor parameters: quantity, typology, detection range, and horizontal field-of-view (FOV). These are detailed in Table 17.

**Figure 7:**
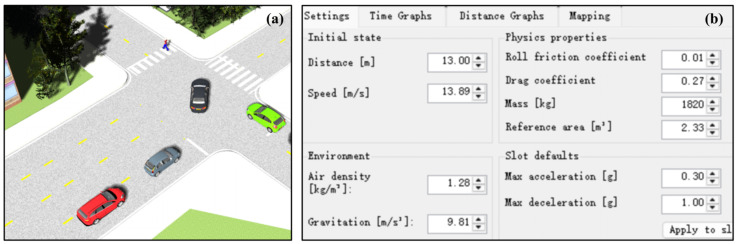
Test scenario and vehicle parameter setting in PreScan 8.5.0: (**a**) test scenario setting and (**b**) vehicle parameter setting.

**Figure 8:**
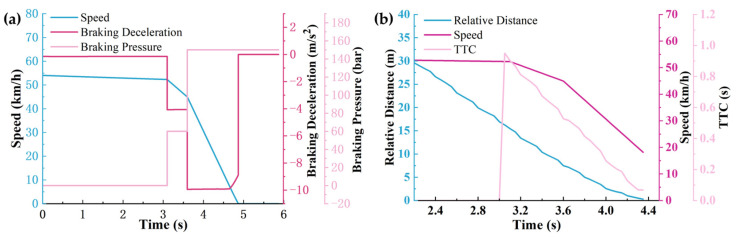
The simulation results under sensor scheme 1: (**a**) The relationship between vehicle speed, brake deceleration, and brake pressure over time. (**b**) The relationship between braking distance, vehicle speed, and warning time over time.

**Figure 9:**
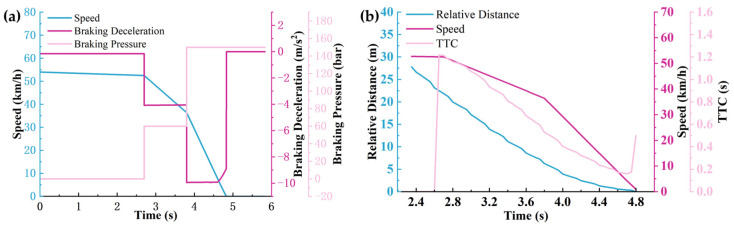
The simulation results under sensor scheme 2: (**a**) The relationship between vehicle speed, brake deceleration, and brake pressure over time. (**b**) The relationship between braking distance, vehicle speed, and warning time over time.

**Figure 10:**
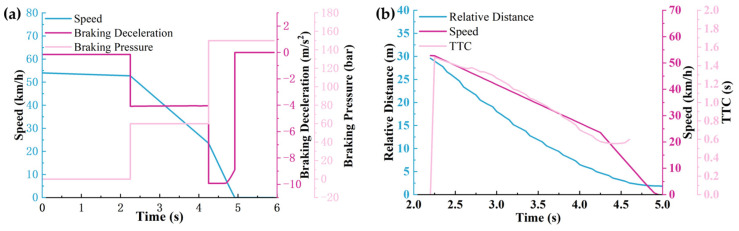
The simulation results under sensor scheme 3: (**a**) The relationship between vehicle speed, brake deceleration, and brake pressure over time. (**b**) The relationship between braking distance, vehicle speed, and warning time over time.

The working conditions and scenario settings are shown in Figure 7, and the simulation results are shown in Figure 8, Figure 9 and Figure 10.

According to Figure 8, Figure 9 and Figure 10, it can be seen that the warning time, partial braking intervention time, and full braking intervention time and duration are inconsistent for schemes 1, 2, and 3. The warning time was 3.05 s, 2.65 s, and 2.25 s, respectively. The duration of the partial braking intervention was 0.5 s, 1.1 s, and 2 s, respectively. The full braking intervention time was 3.6 s, 3.8 s, and 4.25 s, and the intervention duration was 1.26 s, 0.97 s, and 0.67 s, respectively.

Collision avoidance failures were recorded in sensor schemes 1 and 2, characterized by non-zero residual velocities (more than 0 km/h) at zero relative distance thresholds. Conversely, scheme 3 demonstrated successful collision mitigation, achieving complete vehicle rest (0 km/h) while maintaining positive separation distances. Comparative analysis revealed significant variance in collision velocities between schemes 1 and 2, measuring 18.16 km/h and 0.95 km/h, respectively. This velocity discrepancy directly correlates with varying injury severity levels in collision scenarios, suggesting that collision severity modulation through sensor configuration optimization constitutes a critical safety engineering consideration.

Significant variations in obstacle detection latency were observed across different sensor configuration schemes, as evidenced by the experimental data. Early warning initiation was recorded under long-range sensor configurations characterized by extended detection distances (150 m) and restricted horizontal fields of view (<30°). Under these conditions, sub-optimal braking phase coordination was identified, manifesting as abbreviated partial braking durations (<0.5 s) coupled with prolonged full braking intervals (>4.25 s). Furthermore, dynamic target motion trajectories were found to be inaccurately estimated due to horizontal FOV limitations, resulting in emergency collision mitigation failures. This operational deficiency was conclusively linked to compromised safety margins in AEB system performance during critical scenarios.

### 4.3. Analysis of the Main Influencing Factors

**Figure 11:**
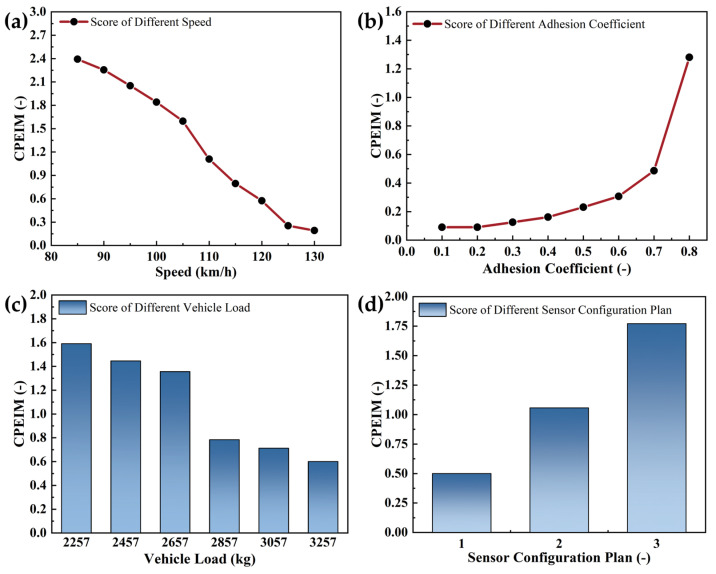
Scores in CPEIM when different influencing factors change: (**a**) Different vehicle speed. (**b**) Different adhesion coefficient. (**c**) Different vehicle load. (**d**) Different sensor configuration.

According to the constructed CPEIM and AEB system scoring criteria, the influence of vehicle load, vehicle speed, road adhesion coefficient, and sensor configuration on the performance of the AEB system in typical test scenarios were analyzed. The simulation results are shown in Figure 11.

As demonstrated in Figure 11, the performance of the AEB system exhibited significant sensitivity to three critical variables: vehicle speed, road adhesion coefficient, and sensor configuration. Quantitatively distinct CPEIM scores were observed when these parameters deviated from baseline values. Specifically, elevated vehicle speeds and reduced adhesion coefficients synergistically diminished braking efficiency, leading to unavoidable collisions under low-adhesion conditions and consequent reductions in collision avoidance rates, which were directly reflected in lower CPEIM scores. The environmental perception capabilities of the AEB system were predominantly constrained by sensor configuration limitations. Suboptimal sensor suites delayed hazard detection in emergency scenarios, resulting in truncated warning intervals and insufficient braking duration. These operational deficiencies precipitated a decline in system reliability indices and substantial performance score variability. While light vehicles demonstrated minimal load-dependent braking distance variance, heavy-duty trucks exhibited significant performance discrepancies between unloaded and fully loaded states. Consequently, this highlights the necessity for adaptive control algorithms in commercial vehicle applications.

In summary, the main factors affecting the performance of the AEB system were vehicle speed, adhesion coefficient, and sensor configuration for light vehicles. But for medium and heavy-duty trucks, they mainly included vehicle speed, adhesion coefficient, sensor configuration, and vehicle load. Therefore, vehicle usage should also be considered when studying and optimizing control strategies for AEB systems.

## 5. Real Vehicle Testing

The efficacy of the CPEIM was validated through real-world road testing involving Tesla and Volvo production vehicles. A representative testing framework was subsequently developed by integrating critical influencing factors (e.g., speed and light limitation) and empirically validated driving scenarios (e.g., pedestrian crosswalk intrusions), enabling a multi-dimensional evaluation of safety performance and functional reliability across heterogeneous AEB system architectures.

### 5.1. Construction of the Road Test Scenarios

The proportion of road participants, road characteristics, and ambient light in daily driving were considered. Typical test scenarios 2 and 4 were used as the main road test scenarios, and the real vehicle testing conditions and scenario settings are shown in Table 18. The experimental field tests were conducted at the Shandong Jiaotong University (Changqing Campus) Intelligent Connected Vehicles Proving Ground, a dedicated facility equipped with Vehicle-to-Everything (V2X) communication base stations. The test zone encompasses standardized road configurations including 800 m straightaways, curved sections, signalized intersections, and gradient variations. All test surfaces are composed of asphalt pavement.

Testing schedules were strictly regulated, with daytime trials at 14:00–16:00 Beijing Time (UTC+8), with ambient illuminance, and nighttime trials at 19:00–21:00 Beijing Time (UTC+8). Meteorological conditions were maintained within good parameters (ambient temperature: 15 ± 2 °C; relative humidity: 25 ± 5%; and wind speed < 3 m/s) throughout the testing period. To ensure experimental consistency, identical driver–vehicle configurations were preserved across all test scenarios.

**Figure 12:**
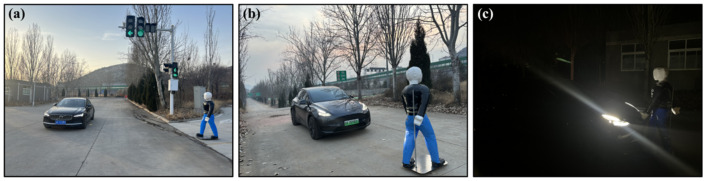
Road test scenarios: (**a**) Pedestrians crossing the intersection. (**b**) Pedestrians crossing the sloping road surface. (**c**) Road V–P reverse direction without streetlights at night.

The Tesla Model Y and Volvo S90 were selected as testing vehicles with differences in sensor configurations. Their assisted driving configurations were shown in Table 19. The target object was a movable adult dummy, with surface characteristics similar to those of real pedestrians. All components can be sensed by sensors, which can simulate the movement state of pedestrians more realistically and meet various test scenarios to ensure testing effectiveness. The road test scenarios are shown in Figure 12.

**Figure 13:**
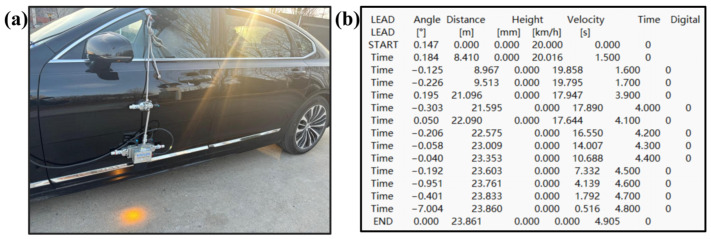
Installation position of the testing system and the data reading from the software: (**a**) Installation location of the testing system. (**b**) The data reading from the software.

The data acquisition employed a Kistler automotive dynamic performance measurement system, mounted externally on the vehicle body with 350 mm vertical ground clearance. Featuring compact dimensions (200 × 100 × 60 mm), lightweight construction (2.3 kg), and environmental interference immunity, its parameter configuration is detailed in Table 20 and the installation position was 350 mm longitudinally from the ground. After the calibration of the testing system, the USB interface data line was used to connect the computer software CeCalWin Pro 1.9.13 to read and save the data for longitudinal speed, acceleration, and driving distance. Each test scenario and operating condition was tested twice. If there was a significant deviation between the two tests, a third test was conducted. Finally, the testing data were comprehensively analyzed in conjunction with the driving recorder. The installation position of the testing system and the data reading from the software are shown in Figure 13.

### 5.2. Analysis of the Road Testing Results

Based on the established road test conditions, experiments were conducted under varying parameters, including vehicle speed, light condition, and road gradient. Subsequently, the performance of the AEB system in two test vehicles was evaluated using a custom-developed comprehensive evaluation model and predefined scoring criteria. The experimental results across all test scenarios are systematically summarized in Table 21 and Table 22.

**Figure 14:**
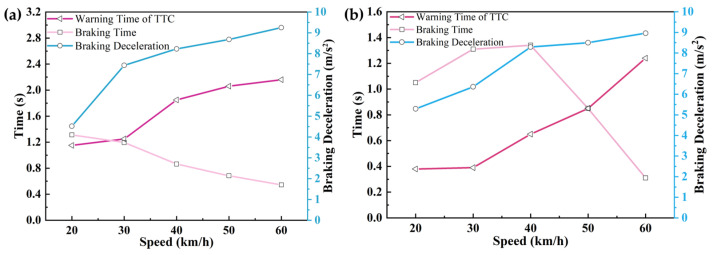
Road testing results under test scenario 1 conditions: (**a**) Tesla Model Y road testing results. (**b**) Volvo S90 road testing results.

**Figure 15:**
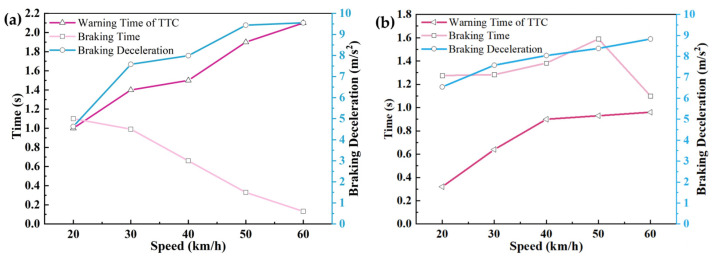
Road testing results under test scenario 2 conditions: (**a**) Tesla Model Y road testing results. (**b**) Volvo S90 road testing results.

**Figure 16:**
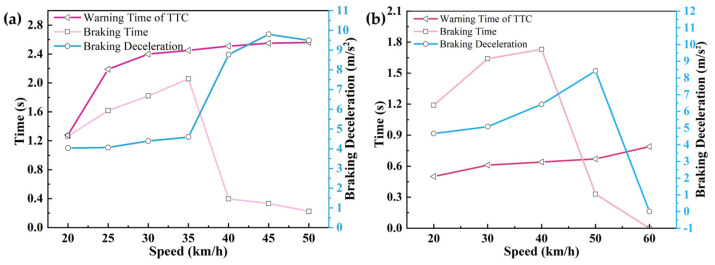
Road testing results under test scenario 3 conditions: (**a**) Tesla Model Y road testing results. (**b**) Volvo S90 road testing results.

The changes in warning time, braking time, and braking deceleration at different vehicle speeds in each scenario are shown in Figure 14, Figure 15 and Figure 16.

As demonstrated in Table 15 and Table 16 and Figure 14, Figure 15 and Figure 16, a progressive increase in AEB system warning intervals was observed with ascending velocity, while intervention braking duration exhibited inverse proportionality. This inverse relationship resulted in diminished average braking deceleration and reduced speed variations. Collision mitigation failures were systematically recorded at velocities exceeding 55 km/h, correlating with compromised vehicular safety indices and degraded driver confidence metrics.

Through CPEIM evaluation, the Tesla Model Y and Volvo S90 were quantified with performance scores of 1.8857 and 2.0433, respectively. Superior early obstacle detection capabilities were demonstrated by the Tesla Model Y across all test scenarios, enabling consistent collision avoidance below 35 km/h. Comprehensive warning and braking coordination was maintained throughout the velocity spectrum.

The Volvo S90 exhibited abbreviated warning intervals but achieved collision prevention thresholds up to 45 km/h, augmented by an integrated seat belt pre-tensioning mechanism that enhanced occupant safety coefficients while maintaining ergonomic comfort. However, degraded performance stability and collision mitigation efficacy were observed in scenario 3 at velocities exceeding 50 km/h.

## 6. Conclusions

To systematically assess the comprehensive performance of AEB systems, the CPEIM was developed, and its effectiveness was validated through multi-vehicle real-world testing. The principal findings are as follows:

1. By means of a K-means clustering analysis of traffic accident data, four characteristic test scenarios were identified:
  - Highway rear-end collisions (V–V type) under adverse weather conditions.
  - V–VRU conflict at intersections and ghost probe incidents on speed-restricted urban roads.
  - Pedestrian/cyclist crossing accidents during inclement weather in urban areas.
  - Rural nighttime collisions (V–P type) under unilluminated road conditions.
2. The CPEIM framework, developed through the AHP, systematically incorporated multidimensional factors including road surface characteristics, vehicular operational parameters, sensor configuration specifications, and AEB system safety-reliability metrics. The five novel comprehensive evaluation indices, including braking parking distance, braking deceleration, collision warning time, speed variation, and accident collision avoidance rate were established in this paper. A methodological integration of international AEB assessment protocols (e.g., Euro NCAP and China NCAP) was implemented to derive scenario-specific evaluation criteria, ensuring alignment with global technical standards while maintaining contextual adaptability.
3. The co-simulation model was built based on MATLAB/Simulink 2020a and PreScan 8.5.0, and the main factors affecting the comprehensive performance of the AEB system were analyzed in typical test scenarios. Moreover, simulation studies revealed three critical performance determinants: initial vehicle velocity, road surface friction coefficient, and sensor configuration parameters, which constitute essential references for standardized test condition development.
4. Real-world validation trials with Tesla Model Y and Volvo S90 in characteristic scenarios yielded the following findings:
  - CPEIM evaluation scores: 1.8857 (Tesla Model Y) and 2.0433 (Volvo S90).
  - Collision avoidance operational thresholds under dry pavement conditions: ≤ 35 km/h (Tesla Model Y) and ≤ 45 km/h (Volvo S90).

These empirical results demonstrated the model’s effectiveness in cross-validating AEB system performance across heterogeneous test environments.

This study is limited by a relatively small accident sample size and the lack of nationally representative traffic data; future research could leverage nationwide traffic accident databases and naturalistic driving data to analyze driving scenario characteristics and construct more comprehensive test scenarios. Furthermore, the co-simulation framework employed to evaluate AEB system performance utilized a single control model architecture, which may restrict the generalizability of the findings. Future research could integrate emerging risk scenarios such as V2X communication and extreme weather conditions to further validate the applicability of the proposed model in commercial vehicles and specialized vehicle categories (e.g., unmanned delivery vehicles).

## Figures and Tables

**Table 1:** Analysis table of scenario element variables.

Scenario Element	Variable Information	Parameter	Number
Weather condition	Weather condition	Clear day	1
		Rain, snow, and other bad weather	2
Light condition	Time and location	Daytime	1
		Night (with street light)	2
		Night (no street light)	3
Main vehicle information	Motion state	Go straight	1
		Turning	2
Target information	Type	Motor vehicle	1
		Two-wheelers	2
		Pedestrian	3
	Motion state	Go straight	1
		Turning	2
	Relative movement with the main vehicle	Same direction	1
		Reverse direction	2
		Vertical	3
	Road type	Straightaway	1
		Intersection (with traffic lights)	2
		Intersection (no traffic light)	3
	Road classification	Urban road	1
		Highway	2
		Township road	3

**Table 2:** The statistical distribution of scenario element constituent ratios.

Variable Information	Number		
	1	2	3
Weather condition	60%	40%	0
Time and location	66%	20%	14%
Main vehicle motion state	72%	28%	0
Target type	6%	68%	26%
Target motion state	18%	82%	0
Relative movement with the main vehicle	19%	9%	72%
Road type	26%	74%	0
Road classification	34%	25%	41%

**Table 3:** The final cluster centroids.

Variable Information	Clustering			
	1	2	3	4
Weather condition	2	1	1	2
Time and location	1	3	1	1
Main vehicle motion state	2	1	1	1
Target type	2	2	2	1
Target motion state	1	1	2	1
Relative movement with the main vehicle	3	1	3	1
Road type	2	1	1	1
Road classification	1	3	1	2

**Table 4:** The distance between the final cluster centers.

Clustering	1	2	3	4
1	0	2.728	2.313	1.96
2	2.728	0	1.588	2.308
3	2.313	1.588	0	2.904
4	1.96	2.308	2.904	0

**Table 5:** The ANOVA table.

Variable Information	Clustering		Error		F	*p*	η^2^
	Mean Square	Degrees of Freedom	Mean Square	Degrees of Freedom			
Weather condition	1.276	3	0.246	21	11.12	<0.001	0.913
Time and location	2.61	3	0.568	21	51.075	<0.001	0.894
Main vehicle motion state	1.312	3	0.11	21	11.962	<0.001	0.882
Target type	1.228	3	0.348	21	10.655	<0.001	0.821
Target motion state	1.112	3	0.11	21	10.022	<0.001	0.536
Relative movement with the main vehicle	5.786	3	0.099	21	58.318	<0.001	0.509
Road type	2.301	3	0.041	21	56.373	<0.001	0.491
Road classification	6.495	3	0.089	21	72.744	<0.001	0.484

**Table 6:** Typical test scenarios for the AEB system.

Category	Scene Participant	Light Condition	Weather Condition	Road Type	Relative Motion	Road Classification
1	V–V	Daytime	Bad weather	Straight	Syntropy	Highway
2	V–VRU ^1^	Daytime	Clear day	Intersection	Vertical	Urban road
3		Daytime	Bad weather	Intersection	Vertical	Urban road
4	V–P	Night (no street light)	Clear day	Straight	Reverse	Country road

^1^ VRU mainly refers to electric two-wheeler riders and conventional bicycle riders in this table.

**Table 7:** The layers based on the AHP for the CPEIM of the AEB system.

Target Layer	Standard Layer	Scheme Layer
The CPEIM of AEB system	Typical test scenario 1 Typical test scenario 2 Typical test scenario 3 Typical test scenario 4	Braking parking distance
		Braking deceleration
		Collision warning time
		Speed variation
		Accident collision avoidance rate

**Table 8:** Important relationship table for AEB system test scenarios.

The Standard Layer	Test Scenario 1	Test Scenario 2	Test Scenario 3	Test Scenario 4
Test Scenario 1	1	1/3	1/4	1/2
Test Scenario 2	3	1	1/2	2
Test Scenario 3	4	2	1	3
Test Scenario 4	2	1/2	1/3	1

**Table 9:** Weight coefficients for each test scenario.

Typical Test Scenario	1	2	3	4
Weight	0.0953	0.2776	0.4668	0.1603

**Table 10:** Weight coefficients of each evaluation index in different typical test scenarios.

Scenario Evaluating Index	Braking Distance	Braking Deceleration	Collision Warning Time	Speed Variation	Accident Collision Avoidance Rate
1	0.1585	0.0965	0.2668	0.0965	0.3817
2	0.1447	0.0901	0.2962	0.0603	0.4087
3	0.1484	0.0903	0.2709	0.0710	0.4194
4	0.1691	0.0790	0.3537	0.0573	0.3409

**Table 11:** Scoring criteria for different vehicle speeds in typical test scenario 2.

Test Scenario	Vehicle Speed (km/h)	Target Speed (km/h)	Scoring Standard					
			Whether to Avoid Collision	Score	Collision Warning Time (s)	Score	Speed Variation (km/h)	Score
Vertical driving of V–VRU at the intersection	20	10	yes	2	1.2 ≤ TTC	1	-	1
	30		yes	1		1	-	1
	40		yes	2	1.4 ≤ TTC	1	More than 20	2
	50		Avoid or reduce collision speed	3		2		2
	60			3	1.8 ≤ TTC	1		1

Note: No scores will be awarded when the warning time is less than the corresponding value and the speed variation is less than 20 km/h.

**Table 12:** Scoring criteria of braking parking distance.

Relative Distance s (m)	0 < s ≤ 0.6	0.6 < s ≤ 1.2	1.2 < s ≤ 1.8	1.8 < s ≤ 2.4	2.4 < s
Score	1	0.8	0.6	0.3	0

**Table 13:** Scoring criteria for braking deceleration.

Vehicle Speed (km/h)	20–30	20–30	20–30	30–50	30–50	30–50	More than 50	More than 50
MFDD (m/s^2^)	MFDD ≤ 2	2 < MFDD ≤ 5.0	5.0 < MFDD	MFDD ≤ 5.0	5.0 < MFDD ≤ 7.0	7.0 < MFDD	MFDD < 6.0	6.0 < MFDD
Score	0	1	0.5	0	1	0.5	0	1

**Table 14:** The model parameters of the AEB system.

Model Parameter	Value Range
Detection range	30–150 m
TTC threshold	1.6 s (partial braking), 0.6 s (full braking)
Deceleration	5–10 m/s^2^
Reaction time	0.2–0.5 s

**Table 15:** The main vehicle dynamics model parameters.

Parameter	Value	Unit
Mass	1820	kg
Reference area	2.33	m^3^
Roll friction coefficient	0.01	-
Distance from center of mass to front axle	1650	mm
Distance from center of mass to rear axle	1645	mm
Wheelbase	3128	mm

**Table 16:** Simulation test scenarios settings.

Simulation Testing Condition	Test Scenario	Testing Vehicle Speed *v_x_* (km/h)	Target Vehicle Speed *v_ob_* (km/h)	Relative Distance ∆*d* (m)	Road Adhesion Coefficient
1	Typical test scenario 1	80–140 (step length 10)	80	10	0.5
2		120	80	15	0.1–1.0
3	Typical test scenario 2	30–80 (step length 10)	10	10	0.9
4	Typical test scenario 3	30–80 (step length 10)	10	10	0.5
5		60	5	10	0.1–1.0
6	Typical test scenario 4	20–60 (step length 10)	5	10	0.5

**Table 17:** Sensor settings.

Scheme	Quantity	Detection Range (m)			Horizontal FOV (Degree)		
		Long Range	Mid-Range	Short Range	Long Range	Mid-range	Short Range
1	3	1*150	/	2*30	20	/	120
2	4	3*150	2*100	/	45	180	/
3	10	7*150	2*100	1*30	105	180	180

Note: Schemes 1 and 2 radars were installed at the front bumper. In Scheme 3, two mid-range radars were installed at the left and right mirrors, three long-distance radars at the interior mirrors, and the rest at the front bumper.

**Table 18:** Setting of testing conditions.

Test Scenario	Speed of the Testing Vehicle (km/h)	Target Speed (km/h)	Light	Road Information	Is There a Slope
1	20–60	5	Daytime	Intersection	No
2		0	Daytime	Straight	Yes
3	20–50	5	Night (no street light)	Straight	No

**Table 19:** Assisted driving configurations of testing vehicles.

Testing Vehicle	Sensor Type	Number of Sensors	Horizontal Field of View (degree)	Sensor Detection Range (m)
Tesla Model Y	Ultrasonic radar, millimeter-wave radar, camera ^1^	21	360	424
Volvo S90	Ultrasonic radar, camera	16	360	200

^1^ The environmental perception technology in the AEB system predominantly employs a vision-only approach.

**Table 20:** Parameter configuration of automotive dynamic performance testing system.

Measured Variable	Range	Accuracy
Forward speed	0.1~70 m/s	<±0.1%
Longitudinal acceleration	±29.4 m/s^2^	±0.1% full range
Lateral acceleration		
Lateral angular velocity	±150°/s	

**Table 21:** Results of Tesla Model Y AEB system performance testing.

Speed (km/h)	Warming	Braking	Whether to Avoid Collision	Collision Speed (km/h)			Relative Distance (m)		
				Test Scenario 1	Test Scenario 2	Test Scenario 3	Test Scenario 1	Test Scenario 2	Test Scenario 3
20	√	√	√	0	0	0	1.1	1.7	0.5
30	√	√	√	0	0	0	1.1	1.65	0.1
40	√	√	×	17	24	22	0	0	0
50	√	√	×	32	32	36	0	0	0
60	√	√	×	49	45	/	0	0	/

**Table 22:** Results of Volvo S90 AEB system performance testing.

Speed (km/h)	Warming	Braking	Whether to Avoid Collision	Collision Speed (km/h)			Relative Distance (m)		
				Test Scenario 1	Test Scenario 2	Test Scenario 3	Test Scenario 1	Test Scenario 2	Test Scenario 3
20	√	√	√	0	0	0	1.2	1.5	0.8
30	√	√	√	0	0	0	1.2	1.1	0.75
40	√	√	√	0	0	0	0.7	0.7	0.65
50	√	√	×	24	2	40	0	0	0
60	√	Test scenario 3 was not braking	×	50	25	/	0	0	/

## Data Availability

Data are contained within the article.

## Appendix A

**Table A1:** Table of relationship importance between scheme layer and test scenario 1.

Test Scenario 1	Braking Distance	Braking Deceleration	Collision Warning Time	Speed Variation	Accident Collision Avoidance Rate
Braking Distance	1	2	1/2	2	1/3
Braking Deceleration	1/2	1	1/3	1	1/3
Collision Warning Time	2	3	1	3	1/2
Speed Variation	1/2	1	1/3	1	1/3
Accident Collision Avoidance Rate	3	3	2	3	1

**Table A2:** Table of relationship importance between scheme layer and test scenario 2.

Test Scenario 2	Braking Distance	Braking Deceleration	Collision Warning Time	Speed Variation	Accident Collision Avoidance Rate
Braking Distance	1	2	1/3	3	1/3
Braking Deceleration	1/2	1	1/4	2	1/4
Collision Warning Time	3	4	1	4	1/2
Speed Variation	1/3	1/2	1/4	1	1/5
Accident Collision Avoidance Rate	3	4	2	5	1

**Table A3:** Table of relationship importance between scheme layer and test scenario 3.

Test Scenario 3	Braking Distance	Braking Deceleration	Collision Warning Time	Speed Variation	Accident Collision Avoidance Rate
Braking Distance	1	3	1/3	2	1/3
Braking Deceleration	1/3	1	1/3	2	1/4
Collision Warning Time	3	3	1	3	1/2
Speed Variation	1/2	1/2	1/3	1	1/5
Accident Collision Avoidance Rate	3	4	2	5	1

**Table A4:** Table of relationship importance between scheme layer and test scenario 4.

Test Scenario 4	Braking Distance	Braking Deceleration	Collision Warning Time	Speed Variation	Accident Collision Avoidance Rate
Braking Distance	1	3	1/3	3	1/2
Braking Deceleration	1/3	1	1/4	2	1/5
Collision Warning Time	3	4	1	5	1
Speed Variation	1/3	1/2	1/5	1	1/5
Accident Collision Avoidance Rate	2	5	1	5	1

**Table A5:** Scoring criteria for different vehicle speeds in typical test scenario 1.

Test Scenario	Vehicle Speed (km/h)	Target Speed (km/h)	Scoring Standards					
			Whether to Avoid Collision	Score	Collision Warning Time (s)	Score	Speed Variation (km/h)	Score
V–V in the same direction at high speeds	80	80–0 (4 s)	Yes	1	2.0 ≤ TTC	1	-	1
	90		Yes	1		1	-	2
	100		Avoid or reduce collision speed	1	2.6 ≤ TTC	2	More than 20	2
	110			2		2		1
	120			2		1	More than 40	2
	130			1		1		1
	90	80	Yes	1	1.8 ≤ TTC	1	-	2
	100		Yes	1		1	-	2
	110		Yes	2		1	-	2
	120		Avoid or reduce collision speed	2	2.4 ≤ TTC	1	More than 40	2
	130			1		2		1

**Table A6:** Scoring criteria for different vehicle speeds in typical test scenario 3.

Test Scenario	Vehicle Speed (km/h)	Target Speed (km/h)	Scoring Standards					
			Whether to Avoid Collision	Score	Collision Warning Time (s)	Score	Speed Variation (km/h)	Score
Vertical driving of V–VRU at crossroads under adverse weather conditions	20	10	Yes	2	1.8 ≤ TTC	1	-	1
	30		Yes	1		1	More than 10	1
	40		Avoid or reduce collision speed	3	2.0 ≤ TTC	1		2
	50			4		2	More than 20	2
	60			5	2.2 ≤ TTC	2		1

**Table A7:** Scoring criteria for different vehicle speeds in typical test scenario 4.

Test Scenario	Vehicle Speed (km/h)	Target Speed (km/h)	Scoring Standards					
			Whether to Avoid Collision	Score	Collision Warning Time (s)	Score	Speed Variation (km/h)	Score
At night (without streetlights), driving in the opposite direction of V–P on straight roads in rural areas	10	5	Yes	1	1.5 ≤ TTC	1	-	1
	20		Yes	1		1	More than 10	1
	30		Avoid or reduce collision speed	3	1.8 ≤ TTC	2		2
	40			4		2	More than 20	2
	50			5		2		2

**Table A8:** Fitting results under various simulation testing conditions.

Simulation Testing Condition	Fitting Equation	Coefficient of Determination (R^2^)
1	y = 35.07917*x*^2^ − 117.59208*x +* 186.22839	0.99748
2	y = 22.43911*x*^2^ − 112.22532*x +* 225.81301	0.99995
3	y = −74.10736*x*^3^ + 247.18262*x*^2^ − 324.79359*x* + 177.07202	0.99993
4	y = 159.17361 × 0.13885*^x^* + 11.45359	0.99913
5	y = −158.97669*x +* 154.32733	0.99615
6	y = 134.19169 × 0.19965*^x^*+ 2.47111	0.99973
